# Better at home: A quality improvement initiative to increase same day discharge after minimally invasive hysterectomies in gynecologic oncology

**DOI:** 10.1016/j.gore.2026.102136

**Published:** 2026-06-15

**Authors:** Pamela N. Peters, Madeline Morello, Jamie Tyler-Walker, Emily Sterrett, Heather McLean, Laura J. Havrilesky

**Affiliations:** a Sutter East Bay Medical Group, Berkeley, CA, United States of America; b Department of Obstetrics and Gynecology, Duke University, Durham, NC, United States of America; c Department of Pediatrics, Duke University, Durham, NC, United States of America

**Keywords:** Minimally invasive hysterectomy, Same day discharge, Void trial, Ambulatory surgery

## Abstract

**Objective:**

To increase the rate of same-day discharge after minimally invasive hysterectomy.

**Methods:**

A quality improvement initiative was implemented at a large academic medical center for patients undergoing minimally invasive hysterectomy by a gynecologic oncologist. The Institute for Healthcare Improvement (IHI) Model for Improvement framework was utilized to define the primary aim, develop interventions, and designate tracked measures. The primary aim was to increase the rate of same-day discharge after minimally invasive hysterectomy from a 61% baseline rate to over 90% within 8 months. Process measures included improving the percentage of cases scheduled as ambulatory surgeries. Balancing measures included emergency department encounters and time spent in the post-anesthesia care unit (PACU). Statistical process control charts were used to track measures. Our main interventions were: 1) creation of standard work (elimination of void trials, standard language in pre-operative surgical consent); 2) patient education initiative; 3) case discussions at division meetings to promote evidence-based care.

**Results:**

The rate of same-day discharge improved significantly from 61% to 85% over a 1-year period. The percentage of minimally invasive hysterectomy cases scheduled as ambulatory surgery improved from 3.5% to 81%. There was no change in the mean time spent in the PACU or in emergency department encounters within 1 week after surgery.

**Conclusions:**

A quality improvement initiative with simple interventions focused the creation of standard work, patient education, and provider culture significantly improved rates of same day discharge after minimally invasive hysterectomy without increasing post-op emergency room visits or time in the PACU.

## Highlights

• Targeted interventions increased same-day discharge from 61% to 85%.
• Eliminating void trials enabled safe, efficient postoperative discharge.
• There was no increase in ED visits or PACU time after implementation.

## 1 Introduction

The widespread adoption of minimally invasive surgery in gynecologic oncology has improved patient outcomes by decreasing complication rates and expediting postoperative recovery (Walsh et al., 2009; Pickett et al., 2023). Same-day discharge after minimally invasive hysterectomy for benign or malignant conditions is safe and cost-effective (Korsholm et al., 2017; Nahas et al., 2016; Gale et al., 2018; Lee et al., 2016). While rates of same-day discharge after minimally invasive hysterectomy for benign indications approach 90% (Gale et al., 2018; Dedden et al., 2017), those undergoing surgery with gynecologic oncology tend to be older and with more medical comorbidities, and have 40–80% same-day discharge rates (Penner et al., 2015; Gien et al., 2011).

Hospital admissions expose patients to risk of hospital-acquired infection and other complications, and place strain on the healthcare system (*HAIs: Reports and Data: U.S. Centers for Disease Control and Prevention*, 2026; Bongiovanni et al., 2021; Dyas et al., 2022; *Hospitalization: National Center for Health Statistics*, 2026). Hospitalization is occasionally medically indicated after minimally invasive hysterectomy. However, we observed at our institution during 2022–2023 that post-hysterectomy hospitalization occurred regularly, often without indications supported by medical evidence. Additionally, like many hospitals, we were often at capacity, meaning many admitted patients stayed in the post-anesthesia care unit (PACU) until discharge due to lack of available hospital beds.

We therefore created a quality improvement initiative to identify causes of hospitalization after minimally invasive hysterectomy and develop interventions to improve rates of same-day discharge. We aimed to improve our rate of same-day discharge after minimally invasive hysterectomy from a baseline rate of 61% from January 2022–September 2023 to greater than 90% by May 31, 2024.

## 2 Methods

### 2.1 Study setting and cohort

This project took place at a large academic tertiary care center in the USA, with over 300 minimally invasive hysterectomies performed annually by seven gynecologic oncologists. The target cohort included individuals undergoing minimally invasive hysterectomy within the division of gynecologic oncology. The local Institutional Review Board reviewed the project proposal as quality improvement and determined it exempt from human subjects research oversight (Pro00117306).

### 2.2 Preintervention state

**Fig. 1:**
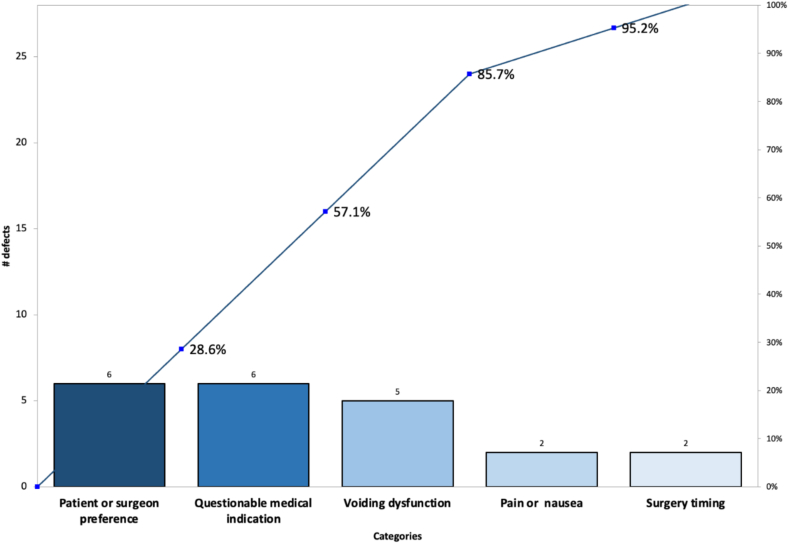
Pareto chart of reasons for non-medically indicated overnight stay after minimally invasive hysterectomy. The three main causes of failure to be discharged the same day of surgery included (Walsh et al., 2009) patient or surgeon preference (Pickett et al., 2023) questionable medical indications and (Korsholm et al., 2017) voiding dysfunction.

The project leader (author PNP), a gynecologic oncology fellow, shadowed frontline teams to create a current state process map (Supplemental Fig. S1) from initial consultation to surgery and discharge. A manual retrospective chart review was performed by the leader to identify common causes of post-hysterectomy hospital admissions. Data were extracted from the electronic medical record (EMR) querying for minimally invasive hysterectomies performed by gynecologic oncologists with postoperative hospitalization for greater than 0 days using CPT codes 58,550, 58552, 58553, 58570, 58571, 58572, 58573, and 58,575 (minimally invasive hysterectomy codes). Charts were manually reviewed to determine reasons for admission. Medically indicated admissions, defined as those with a specific documented reason that required hospital level monitoring (e.g. repeat labwork, continuous oxygen monitoring), were identified. A Pareto chart (Fig. 1) was created to categorize reasons for admission that did not clearly fall under the category of medically indicated, to identify the top three key categories:

1) Patient or surgeon preference. Patients were occasionally admitted overnight with a documented reason of patient preference or surgeon preference.
2) Medical indications not clearly supported by evidence. Patients were placed into this category when they had a documented reason for admission not clearly supported by evidence and/or not be widely accepted as a medical indication for admission. Common examples included advanced age in the absence of other significant medical comorbidities, and well-controlled obstructive sleep apnea (OSA). While some patients with OSA require admission after surgery, consensus guidelines support ambulatory surgery in patients with optimized comorbid medical conditions and compliance with continuous positive airway pressure (CPAP) devices (Joshi et al., 2012). Similarly, advanced age alone is not generally considered a contraindication to same-day discharge after minimally invasive surgery (Hansen et al., 2020).
3) Voiding dysfunction. Prior to this project, the gynecologic oncology division utilized a standard protocol requiring patients to void in the PACU prior to discharge. In this protocol, the bladder was filled with 200 mL of saline in the operating room at completion of the surgical case, and the foley catheter was removed immediately afterward. Patients were required to void prior to discharge, and if they were unable to void or voided <100 mL, a standard protocol with bladder scans and possible catheterization or foley replacement was utilized. Due to the additional time required to assess adequate voiding or need for foley teaching, patients were occasionally admitted overnight. The use of intra-operative cystoscopy was surgeon-dependent and not dictated by a division-wide protocol.

We developed interventions targeting these drivers of overnight stay by focusing on patient expectations and preparation, creation of standard work, and promotion of a culture of evidence-based care.

### 2.3 Working group

A multidisciplinary working group was developed. The project team leader was sponsored by multidisciplinary physician leaders in quality and safety. Additional members included gynecologic oncologists, anesthesiologists (inpatient and pre-operative clinic), advance practice providers (APPs) in the division of gynecologic oncology, nurses (outpatient gynecologic oncology, outpatient anesthesia, and inpatient pre and post operative), administrators, and a quality and safety manager.

### 2.4 Measures and aims

The patient population was defined as patients undergoing minimally invasive hysterectomy with gynecologic oncology at a single site (Duke University Hospital). This population was defined by minimally invasive hysterectomy CPT codes performed by gynecologic oncologists as designated above. Concomitant procedure codes (such as lymphadenectomy, sentinel lymph node biopsy, or tumor debulking procedures) were allowed. Patients were seen for pre-operative evaluation at two clinics. Same-day discharge was defined as prior to midnight on the day of surgery.

The primary outcome measure, the rate of same-day discharge after minimally invasive hysterectomy, was 61% during the baseline period (January 2022–September 2023). In the absence of national benchmarks, reasonable short-term goals were chosen by analyzing internal data to understand the frequency of medically indicated overnight stay after hysterectomy. We found a baseline rate of 9.8% for admissions with documented medical indications. As a result, we set a goal of same day discharge rate of 90%. Our primary goal was therefore to improve our rate of same day discharge after minimally invasive hysterectomy at our institution from a baseline rate of 61% to greater than 90% by May 31, 2024 (Table 1), to correspond with data collection past conclusion of an academic year. Data were collected in a continuous fashion and analyzed longitudinally per best practices in quality improvement, using annotated control charts.

**Table 1:** Project measures with definitions.

Type	Measure	Operational definition	Baseline rate	Goal
Primary outcome	Percentage of patients discharged home on the same day of surgery	N: number of patients discharged the same day after minimally invasive hysterectomy D: number of patients undergoing minimally invasive hysterectomy	61%	To increase from 61% to 90% by May 31, 2024.
Process	Percentage of cases booked as ambulatory surgery	N: number of patients undergoing minimally invasive hysterectomy booked as ambulatory surgery D: number of patients undergoing minimally invasive hysterectomy	3.5%	Increase
Balancing	Percentage of patients with ED visit within 1 week of surgery	N: number of patients undergoing minimally invasive hysterectomy with ED visit within 1 week of surgery D: number of patients undergoing minimally invasive hysterectomy	1.3%	No change
Balancing	Average time spent in PACU per patient	Average time (in hours) from “out of OR” to discharge for patients undergoing minimally invasive hysterectomy with same day discharge	3.6 h	No change

ED, emergency department; D, denominator; N, numerator; PACU, post-anesthesia care unit

Measures, baseline rates, and aims are described in Table 1. Our process measure was the percentage of minimally invasive hysterectomy cases posted as ambulatory surgery. Balancing measures included percentage of patients with emergency department encounters within 1 week after surgery and time spent in the PACU. The time frame of 1 week for emergency department encounters was selected to ensure our change to voiding protocol did not result in an increase in ER visits for acute urinary retention. Time spent in the PACU was defined as time from arrival in the PACU to time of discharge for same-day discharged patients; longer stay patients did not have PACU times available in the EMR because the exact timing of conversion to overnight status was not captured.

### 2.5 Interventions

**Fig. 2:**
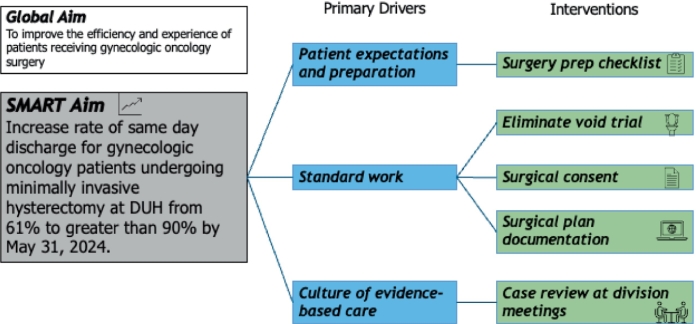
Key driver diagram. The blue boxes represent primary drivers, and the green boxes represent interventions.

We designed our interventions based on a Pareto analysis (Fig. 1) and a pre-intervention process map (Supplemental Fig. 1) outlining the workflow from initial clinic visit to discharge after hysterectomy. We classified key drivers of successful same-day discharge into three categories, and primary interventions were designed to support these key drivers (Fig. 2):

1. Patient expectations and preparation. Patient preference for overnight stay was a top reason for admission. To investigate this in detail prior to designing interventions, phone calls were placed to six patients to assess their satisfaction with their discharge experience. These patients were selected in a semi-random fashion, ensuring we included diverse selection of patients by age, race, and distance from the hospital, as well as mixture patients who were discharged the same day versus stayed overnight. These open-ended phone calls were designed to assess patient satisfaction with their experience in relation to discharge. We identified two primary drivers that would facilitate same-day discharge: 1) clear expectations from medical team regarding discharge plans and 2) adequate support at home. This highlighted a need to improve patient counseling and education on the benefits of same-day discharge, and to clearly inform patients of the plan for same-day discharge to allow patient and family preparation. Interventions designed to address this driver included:
  a. *Patient preparation checklist*. We reviewed our existing pre-operative patient educational materials to confirm accurate information about same-day discharge. After review of these materials, they were translated to Spanish. We created a one-page cover sheet to summarize key information and prepare patients for same-day discharge (Supplemental Fig. 2). This cover sheet was filled out by the clinic nurse with the patient during pre-operative teaching in clinic and indicated whether they were planned for hospital admission or same-day discharge. It asked them to designate a driver and support person for the first night at home.
2. Standard work. We created and modified existing standard work to facilitate the goal of same-day discharge.
  a. *Elimination of void trials.* We reviewed the evidence on void trials after hysterectomy and identified several studies supporting the safety of omitting void trials after hysterectomy (Dong et al., 2022; Chao and Mansuria, 2019; Zakhari et al., 2021; Farag et al., 2021; Moawad et al., 2019). We changed our protocol to foley catheter removal in the operating room without filling the bladder or requirement to void prior to discharge. We reinforced this new workflow with surgeons, trainees, and peri-operative staff. Our standard discharge instructions already included guidance to seek medical attention if unable to void after surgery.
  b. *Standard language in surgical consent.* We adjusted the standard consent language for minimally invasive hysterectomy procedure consents to eliminate “possible exploratory laparotomy”. We had determined in our process mapping that placing this terminology on the top line of the consent administratively required cases to be classified as “inpatient” within the EMR. This led to miscommunication about plans for admission after surgery from pre-operative nursing teams who contact patients by phone the night prior to surgery to discuss arrival time and expectations. By eliminating laparotomy from the surgical consent's top line and the surgical case posting, and moving it to the section describing surgical risks, all procedures were able to be appropriately posted as “ambulatory surgery,” allowing all members of the care team to see and communicate the plan for same-day discharge.
  c. *Standard documentation in pre-op history and physical (H&P).* We added a section to a pre-existing pre-operative H&P template that required providers to indicate the discharge plan (same-day discharge or planned admission), and whether this had been communicated to the patient.
3. Culture of evidence-based care. We created a forum for clinicians to review clinical cases to promote the goal of same-day discharge.
  a. *Case review at division meetings.* During late 2023 and early 2024, we reviewed cases of hospital admissions after minimally invasive hysterectomy at monthly division meetings to engage surgeons and provide opportunities to review evidence-based practices.

### 2.6 Analysis

Data analyses were conducted using Microsoft Excel QI Macros (KnowWare International, Denver, CO). The primary outcome measure, the same-day discharge rate, was tracked over time using a statistical process percentage control chart (p-chart). Special cause variation was evaluated to identify significant changes in this measure over time because of project interventions. The following IHI rules were used to determine special cause variation (Frankel et al., 2017): 1 point above the Upper Control Limit or below the Lower Control Limit, 2 out of 3 points in a row above/below 2 sigma, 8 points in a row above/below the center line, 6 points in a row ascending or descending, and 15 points in a row inside ±1 sigma. Process and balancing measures were tracked over time using run charts. Patient charts of post-operative emergency department encounters were reviewed to determine indication.

## 3 Results

### 3.1 Baseline and demographic data

The baseline rate of same-day discharge after minimally invasive hysterectomy during the pre-intervention period was 61%. Table 2 displays patient demographics in the pre-intervention and post-intervention periods. Age, BMI, distance to the hospital, ethnicity, race, insurance status, and diagnoses were all similar in the pre- and post-intervention periods.

**Table 2:** Characteristics of individuals undergoing minimally invasive hysterectomy with gynecologic oncology.

	Pre-Intervention (1/1/22–9/30/23)	Post-Intervention (10/1/23–10/30/24)
Patients undergoing minimally invasive hysterectomy	596	372
Age (years, mean (SD))	59.0 (13.2)	59.0 (13.4)
Body mass index (kg/m2, mean (SD))	32.6 (8.8)	33.9 (8.9)
Distance to Duke University Hospital (miles, mean (SD))	54.1 (246.1)	49.6 (131.6)
ASA classification (mean (SD))	2.6 (0.5)	2.6 (0.6)
Ethnicity Hispanic Non-Hispanic Not Reported or declined	32 (5.4%) 504 (84.6%) 60 (10.1%)	22 (5.9%) 312 (83.9%) 38 (10.2%)
Race Asian Black/ African American White Other Not reported or declined	19 (3.2%) 130 (21.8%) 380 (63.8%) 21 (3.5%) 46 (7.7%)	13 (3.5%) 89 (23.9%) 234 (62.9%) 13 (3.5%) 23 (6.2%)
Insurance Private Medicare Medicaid Other government	347 (58.2%) 214 (35.9%) 26 (4.4%) 9 (1.5%)	215 (57.8%) 137 (36.8%) 16 (4.3%) 4 (1.1%)
Indication for surgery Non-malignant adnexal pathology Cancer risk reduction Cervical malignancy Endometrial malignancy Endometriosis or adenomyosis Non-gynecologic malignancy Ovarian/fallopian/peritoneal malignancy Premalignant condition of the cervix Premalignant condition of the endometrium Uterine fibroids Uterine or cervical polyp Other	35 (5.9%) 25 (4.2%) 8 (1.3%) 301 (50.5%) 31 (5.2%) 10 (1.7%) 37 (6.2%) 15 (2.5%) 19 (3.2%) 73 (12.2%) 18 (3.0%) 24 (4.0%)	22 (5.9%) 4 (1.1%) 5 (1.3%) 188 (50.5%) 27 (7.3%) 4 (1.1%) 24 (6.5%) 13 (3.5%) 12 (3.2%) 48 (12.9%) 9 (2.4%) 16 (4.3%)

SD, standard deviation; ASA, American Society of Anesthesiologists.

### 3.2 Outcome measure

**Fig. 3:**
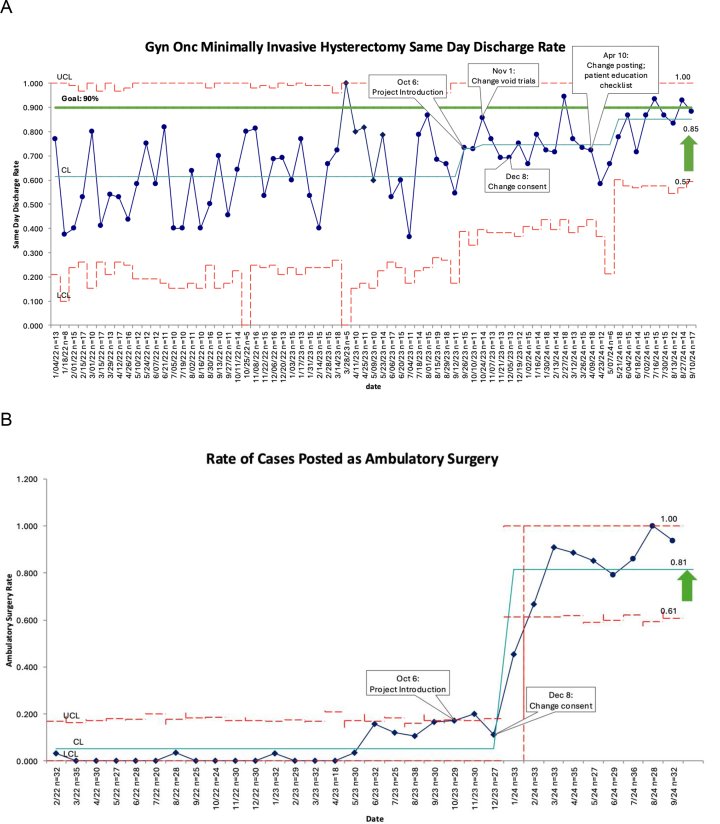
Statistical control charts for outcome, process, and balancing measures for individuals undergoing minimally invasive hysterectomy with gynecologic oncology. (A) Outcome measure, percentage of patients with same day discharge undergoing minimally invasive hysterectomy (B) Process measure, percentage of cases posted as ambulatory surgery.

The rate of same-day discharge after minimally invasive hysterectomy showed statistically significant improvement resulting in a shift of the centerline, meeting criteria for special cause variation (Fig. 3A). By September 2024, the rate of same-day discharge was 85%. While below the target rate of 90%, this improvement was sustained beyond the May 31, 2024 project endpoint.

### 3.3 Process measure

The process measure of percentage of cases posted as ambulatory surgery showed improvement from a baseline of 3.5% of cases to 81% of cases resulting in a shift in the centerline, meeting criteria for special cause variation (Fig. 3B).

### 3.4 Balancing measures

The rate of ED encounters within 1 week after minimally invasive hysterectomy was 1.3% at baseline and remained unchanged following interventions (Supplemental Fig. 3). We reviewed patient charts for all ED encounters within 1 week of surgery and determined the indication for the visit (Supplemental Table 1). Most patients (60%) who presented to the ER within one week of surgery had been observed overnight after their hysterectomy, while 40% had been discharged on the same day. Time spent in the PACU was 3.6 h at baseline and did not change after the intervention (Supplemental Fig. 4).

### 3.5 Implementation

As interventions were designed, Plan-Do-Study-Act (PDSA) cycles were used to evaluate whether they resulted in the intended outcomes (Supplemental Table 2). PDSA cycles were utilized to test the elimination of void trials, the updated surgical consent, a new surgical plan checklist, and the patient education checklist. Before fully implementing each intervention, feedback was gathered from each test (“Study”) and the project team decided to fully adopt, modify and scale up, or abandon the intervention. All interventions except the surgical plan checklist required modifications before rollout to the entire division.

## 4 Discussion

This quality improvement project improved the rate of same-day discharge after minimally invasive hysterectomy from 61% to 85% over a 1-year period. This improvement was achieved in a gynecologic oncology patient population with a median age of 59 and multiple medical comorbidities, demonstrated by 62% with ASA score 3 or higher. Our interventions were straightforward and were focused on process standardization, patient and provider education. Although our goal was 90%, baseline analysis suggested approximately 10% of patients had medically indicated reasons for overnight observation. As a result, same-day discharge rate substantially above 90% would likely require more resource-intensive interventions addressing additional social, logistical, and patient specific factors. Importantly, our initiative was not intended to eliminate clinically appropriate admissions. Surgeons retained discretion to admit patients when post-operative monitoring was warranted. Instead, we sought to reduce unwanted variation in admission practice by reviewing cases at department meetings in which the need for overnight admission was uncertain, and promote evidence-based decision making. Reducing unnecessary hospitalization is important for health systems faced with staffing and bed shortages, as well as reduced risk of hospital acquired infection and other iatrogenic risks for individual patients. (*HAIs: Reports and Data: U.S. Centers for Disease Control and Prevention*, 2026; Bongiovanni et al., 2021; Dyas et al., 2022; *Hospitalization: National Center for Health Statistics*, 2026; Leuchter et al., 2025). Also importantly, the same-day discharge rate improved without increasing emergency department encounters during the first postoperative week.

This project supports the omission of void trials in the post-operative gynecologic oncology patient population, consistent with prior studies in a benign gynecology population (Dong et al., 2022; Chao and Mansuria, 2019; Zakhari et al., 2021; Farag et al., 2021; Moawad et al., 2019). Void trials after hysterectomy are part of surgical dogma, but evidence suggests that void trials often detect subclinical urinary retention leading to unnecessary catheterization (Dong et al., 2022; Chao and Mansuria, 2019; Zakhari et al., 2021; Farag et al., 2021; Moawad et al., 2019). While clinically significant urinary retention can occur post-hysterectomy, void trials do not seem to be effective to detect or prevent this (Dong et al., 2022). Omission of void trials after hysterectomy appears to be safe in the gynecologic oncology population and can support the goal of same day discharge.

This study demonstrates the importance of detailed observation of an existing system prior to designing interventions. There are several well-described risk factors for hospitalization after minimally invasive hysterectomy in the literature, including surgery timing, surgery duration, opioid use, anesthesia factors, and social factors (Kim et al., 2022; Manorot et al., 2024). While review of the evidence provided our team with important background knowledge, detailed observation of institution-specific processes was essential in guiding our interventions. By creating a process map of our surgical scheduling process and directly observing phone calls from pre-operative nurses to patients, we discovered that consent-based classification of cases during scheduling was a key driver of miscommunication of patient discharge expectations. This allowed us to identify and eliminate an “inpatient” case posting as the root cause of incorrect communications to patients about discharge. While our interventions were focused primarily on features of the process that we could control, observing all parts including those outside of our direct control helped in the design of targeted interventions.

Strengths of this study include the application of low-cost, likely cost-saving interventions, such as omission of void trials, that can be reproduced at other institutions. We were able to track ED encounters through the EMR, leveraging the existing close follow up provided by our institutional National Surgical Quality Improvement Program (NSQIP) abstractor team. This generated robust data that suggested our protocol changes with respect to void trials did not raise a safety concern. We estimate based on the cost of nursing services alone (average of $566–$1132 for an additional 12–24 h of nursing care at our institution), that the same day discharge of 50 additional patients per year in our surgical division will save the hospital $28,300–$56,600 per year in nursing care costs alone.

Study limitations included a lack of quantitative patient satisfaction measures, inability to track PACU times for all patients, and limited ability to track outside ED encounters. Additionally, we did not evaluate whether specific concomitant procedures influenced discharge outcomes. We investigated patient satisfaction surveys already in place at our institution, however these were completed at low rates and did not contain the details needed to guide our interventions. Accurate PACU times were only available for patients discharged the same day, however this was still a useful measure as this EMR limitation existed both pre- and post-intervention. We identified all ED encounters at our hospitals but also relied on our NSQIP team who capture outside ED encounters; NSQIP audits approximately 60% of annual hysterectomy cases at our institution and would include ED visits outside of our institution, allowing us to capture ED visits for many of our patients who live significant distance from our hospital. However, because this represents a sample, it remains possible that some ED encounters were missed.

We were unable to address social barriers to same day-discharge. Patient preference to remain in hospital overnight is multifactorial, but a primary driver is often lack of social support (family, transportation, housing). We did not evaluate area-level socioeconomic factors such as neighborhood income, educational attainment that may also influence the feasibility of same-day discharge. Future work could incorporate this geographic and socioeconomic data to better identify patients at risk for unsuccessful same-day discharge and design targeted intervention. We considered implementation of a screening tool for social barriers to same-day discharge, with connection to support services prior to surgery. However, due to resource limitations within our health system, it was not feasible to intervene on all positive screens. We were able to target patients with increased vulnerability due to age, race, and distance from the hospital in our phone calls to inform design of interventions.

Regarding generalizability, our patient population had a low percentage of uninsured and Medicaid patients, suggesting that our interventions may not be generalizable to other systems with a higher proportion of Medicaid patients. The generalizability of our study may also be limited by the fact that several of our interventions, such as changes to surgical consent, were specific to processes at our institution.

In conclusion, we have used quality improvement methodology to design simple interventions focused on standard work, patient education, and department culture that improved our rate of same-day discharge for minimally invasive hysterectomies from 61% to 85% in 1 year.

## CRediT authorship contribution statement

**Pamela N. Peters:** Writing – review & editing, Writing – original draft, Project administration, Methodology, Investigation, Formal analysis, Data curation, Conceptualization. **Madeline Morello:** Writing – review & editing, Visualization, Formal analysis, Data curation. **Jamie Tyler-Walker:** Formal analysis, Data curation. **Emily Sterrett:** Writing – review & editing, Supervision, Project administration, Methodology, Conceptualization. **Heather McLean:** Writing – review & editing, Supervision, Methodology, Conceptualization. **Laura J. Havrilesky:** Writing – review & editing, Writing – original draft, Supervision, Project administration, Methodology, Conceptualization.

## Declaration of competing interest

The authors declare that they have no known competing financial interests or personal relationships that could have appeared to influence the work reported in this paper.
