# Supplementary material for: Better at home: A quality improvement initiative to increase same day discharge after minimally invasive hysterectomies in gynecologic oncology
Source: Gynecol Oncol Rep. 2026 Jun 15;66:102136. doi: 10.1016/j.gore.2026.102136 (PMC13312569; doi:10.1016/j.gore.2026.102136)
Supplement: Supplementary material 1 [file mmc1.docx]

**Supplemental Figure 1. Minimally invasive hysterectomy process map: pre-intervention state**

MIS, minimally invasive; APP, advanced practice provider; RN, registered nurse; PASS, pre-operative Anesthesia and Surgical Screening; OR, operating room; PACU, post-anesthesia care unit; CRNA, certified registered nurse anesthetist

**
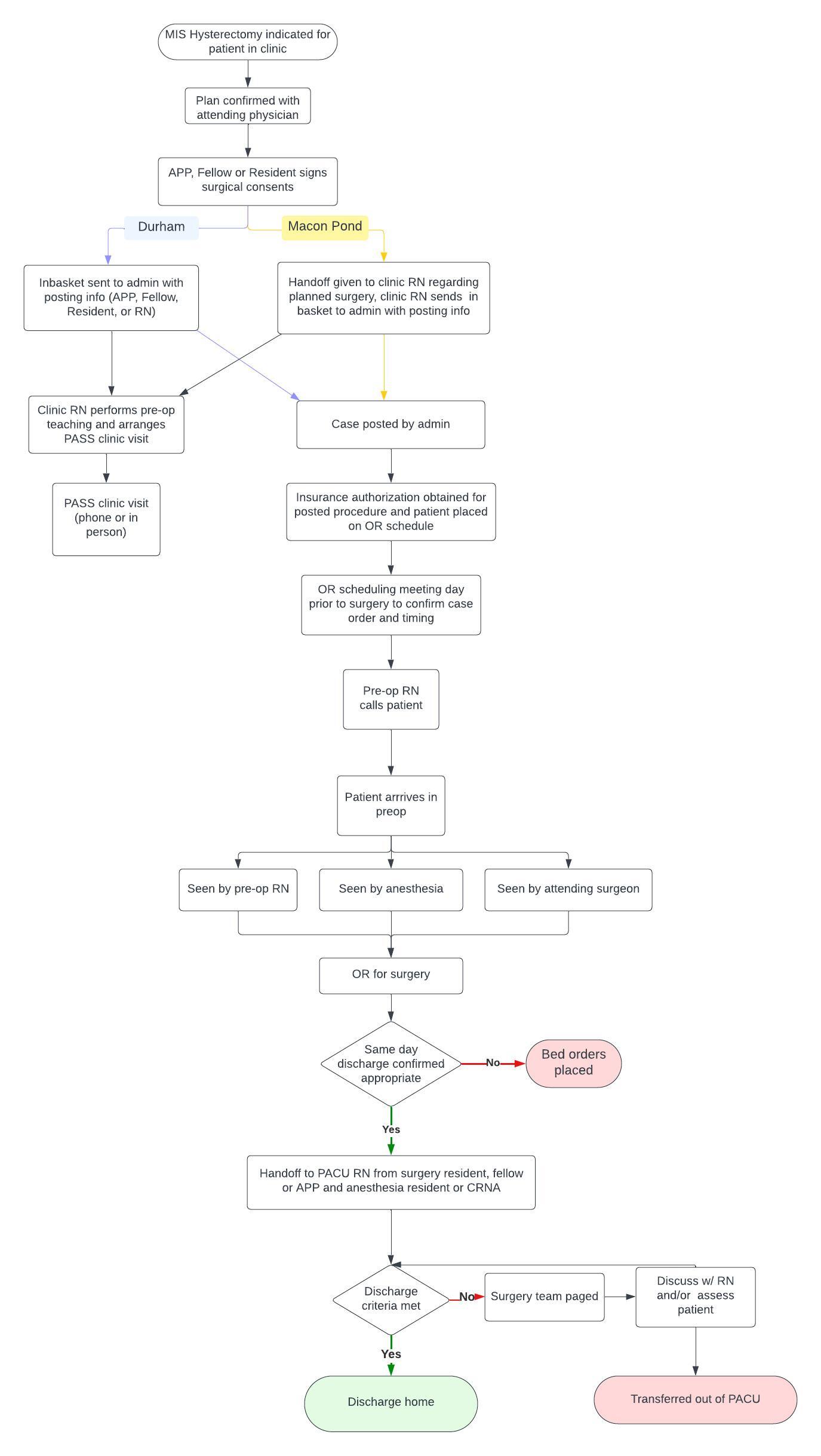
**
