# Supplementary figures and images for: Better at home: A quality improvement initiative to increase same day discharge after minimally invasive hysterectomies in gynecologic oncology
Source: Gynecol Oncol Rep. 2026 Jun 15;66:102136. doi: 10.1016/j.gore.2026.102136 (PMC13312569; doi:10.1016/j.gore.2026.102136)

**Supplemental Figure 2. Patient preparation checklist**

**
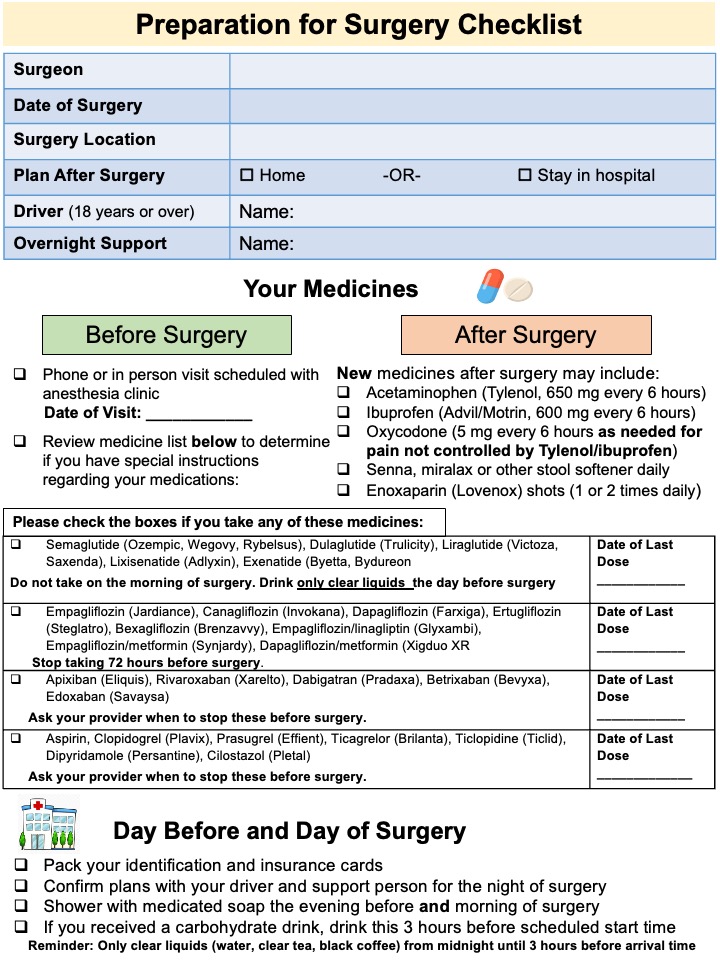
**

Supplement: Supplementary material 2 [file mmc2.docx]
