# Supplementary material for: Better at home: A quality improvement initiative to increase same day discharge after minimally invasive hysterectomies in gynecologic oncology
Source: Gynecol Oncol Rep. 2026 Jun 15;66:102136. doi: 10.1016/j.gore.2026.102136 (PMC13312569; doi:10.1016/j.gore.2026.102136)
Supplement: Supplementary material 3 [file mmc3.docx]

**Supplemental Figure 3.** Balancing measure, percentage of patients with emergency department visit within 1 week of surgery

**
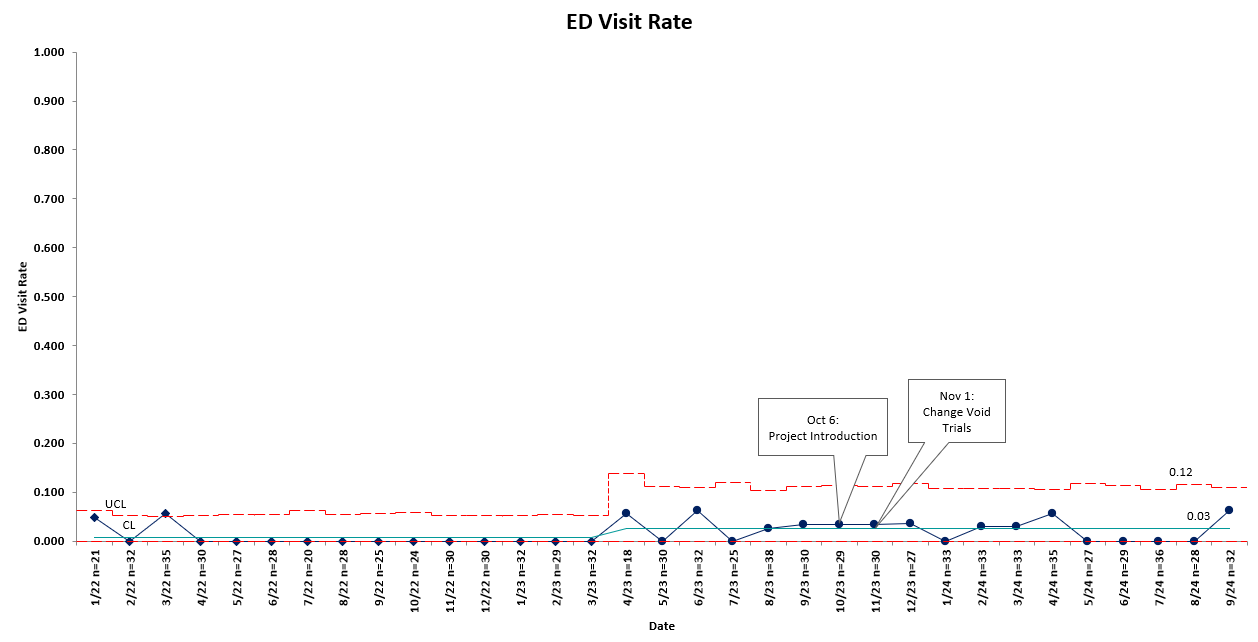
**
