# Supplementary material for: Better at home: A quality improvement initiative to increase same day discharge after minimally invasive hysterectomies in gynecologic oncology
Source: Gynecol Oncol Rep. 2026 Jun 15;66:102136. doi: 10.1016/j.gore.2026.102136 (PMC13312569; doi:10.1016/j.gore.2026.102136)
Supplement: Supplementary material 5 [file mmc5.docx]

**Supplemental Table 1. Emergency Department (ED) encounters within 1 week of discharge**

| Procedure | Age | Diagnosis | Post-op Disposition | Post-op Day | ED Visit Reason | ED Disposition |
| --- | --- | --- | --- | --- | --- | --- |
| Robotic TLH BSO SLND | 70 | Endometrial cancer | Overnight stay (1 day) | 8 | Purulent drainage from incision site | Discharge |
| Robotic TLH BSO SLND | 65 | Endometrial cancer | Overnight stay (1 day) | 4 | Abdominal pain, nausea | Hospital admission |
| TLH BSO | 68 | Adnexal mass, uterine fibroids | Overnight stay (1 day) | 2 | Leukocytosis identified at urgent care | Discharge |
| Robotic TLH BSO SLND | 56 | Endometrial cancer | Same Day Discharge | 2 | Dizziness and blurry vision | Discharge |
| Robotic TLH BSO SLND | 45 | Endometrial intraepithelial neoplasia (EIN) | Same Day Discharge | 4 | Fever and dysuria | Discharge |
| TLH BSO | 64 | Adnexal mass | Overnight stay (1 day) | 4 | Insomnia and delirium | Hospital admission |
| Robotic TLH upper vaginectomy, BSO SLND | 60 | Cervical cancer | Same Day Discharge | 0 | Unable to void; urinary tract injury | Hospital admission, return to OR |
| Robotic TLH BSO SLND | 59 | Endometrial cancer | Overnight stay (1 day) | 4 | Emesis | Hospital admission |
| *TLH BSO SLND | 57 | Endometrial polyp | Same Day Discharge | 6 | Pain and hypertension | Discharge |
| *Robotic TLH BSO | 48 | Uterine fibroids | Overnight stay (1 day) | 3/6  (2 encounters) | Pain | Discharge |
| *Indicates encounters to EDs outside of our institution  ED, emergency department; TLH, total laparoscopic hysterectomy; BSO, bilateral salpingo-oophorectomy; SLND, sentinel lymph node dissection; OR, operating room | | | | | | |
