# Supplementary material for: Better at home: A quality improvement initiative to increase same day discharge after minimally invasive hysterectomies in gynecologic oncology
Source: Gynecol Oncol Rep. 2026 Jun 15;66:102136. doi: 10.1016/j.gore.2026.102136 (PMC13312569; doi:10.1016/j.gore.2026.102136)
Supplement: Supplementary material 6 [file mmc6.docx]

**Supplemental Table 2. Plan-Do-Study-Act (PDSA) cycles with interventions**

| Mini-Aim | Key Driver | PDSA Cycle # | Dates | Population | Lessons Learned | Action |
| --- | --- | --- | --- | --- | --- | --- |
| Introduce project aims and supporting data | Culture of evidence-based care | 1. Establish same day discharge as the standard of care | October 6, 2023 | Gyn oncology surgeons, fellows, and APPs | Surgeons have different thresholds for admission to the hospital and different perspectives on the value of same day discharge | Establish regular review of cases at division meeting to highlight opportunities to improve evidence-based care and continue dialogue within the division |
| Discontinue required void trials in PACU | Standard work | 1. Void trial order set no longer used | November 1, 2023 to present | All patients | Elimination of void trials was widely popular among PACU nurses and there was no increase in ED encounters for urinary retention | Adopt |
| Change standard surgical consent to eliminate “possible laparotomy” from procedure name to risks section | Standard work | 1. Clearly communicate the plan for minimally invasive surgery with low risk of conversion to laparotomy | December 8, 2023 to present | All offices | Elimination of possible laparotomy within the procedure name increased number of cases posted as ambulatory surgery | Adopt |
| Create a “surgical plan” checklist in the standard pre-op H&P | Standard work | 1. Trial new checklist within pre-op H&P including plan for same day discharge | January 23, 2024-January 24, 2024 | Two outpatient clinics at Durham location | The checklist was easy to use, minor changes suggested | Scale up |
|  |  | 2. Trial revised checklist within pre-op H&P including plan for same day discharge | March 25, 2024-March 29, 2024 | One week of outpatient clinics at Durham and Macon Pond locations | Checklist was easy to use and clear, people will utilize if it does not add significantly to documentation burden | Scale up |
|  |  | 3. Change system-wide dot phrase for pre-op H&P to include checklist | April 10, 2024 to present | All offices | Same as above | Adopt |
| Create patient education checklist to promote communication around same day discharge | Patient expectations and preparation | 1. Clinic nurses review checklist with patient as coversheet for GYN ONC pre-op education packets | April 10, 2024 to present | All offices | Use of patient checklist requires that plan for same day discharge or admission is addressed | Adopt |
| Create Spanish language pre-op education packet | Patient expectations and preparation | 1. Implement Spanish language | April 10, 2024 to present | All offices | Creation of a Spanish language version of pre-existing patient education was simple and quick using university-provided resources | Adopt |
